# The Uncoupling Effect of 17β-Estradiol Underlies the Resilience of Female-Derived Mitochondria to Damage after Experimental TBI

**DOI:** 10.3390/life14080961

**Published:** 2024-07-30

**Authors:** Olivia J. Kalimon, Hemendra J. Vekaria, Paresh Prajapati, Sydney L. Short, W. Brad Hubbard, Patrick G. Sullivan

**Affiliations:** 1 Department of Neuroscience, University of Kentucky, Lexington, KY 40508, USA; olivia.kalimon@uky.edu; 2 Spinal Cord and Brain Injury Research Center, University of Kentucky, Lexington, KY 40536, USA; hemendravekaria@uky.edu (H.J.V.); paresh.prajapati@uky.edu (P.P.); sydney.lay@uky.edu (S.L.S.); bradhubbard@uky.edu (W.B.H.); 3 Lexington Veterans Affairs Healthcare System, Lexington, KY 40502, USA; 4 Department of Physiology, University of Kentucky, Lexington, KY 40508, USA

**Keywords:** sex differences, brain trauma, bioenergetics, electron transport chain, neurotherapeutic

## Abstract

Current literature finds females have improved outcomes over their male counterparts after severe traumatic brain injury (TBI), while the opposite seems to be true for mild TBI. This begs the question as to what may be driving these sex differences after TBI. Estrogen is thought to be neuroprotective in certain diseases, and its actions have been shown to influence mitochondrial function. Mitochondrial impairment is a major hallmark of TBI, and interestingly, this dysfunction has been shown to be more severe in males than females after brain injury. This suggests estrogen could be playing a role in promoting “mitoprotection” following TBI. Despite the existence of estrogen receptors in mitochondria, few studies have examined the direct role of estrogen on mitochondrial function, and no studies have explored this after TBI. We hypothesized ex vivo treatment of isolated mitochondria with 17β-estradiol (E2) would improve mitochondrial function after experimental TBI in mice. Total mitochondria from the ipsilateral (injured) and contralateral (control) cortices of male and female mice were isolated 24 h post-controlled severe cortical impact (CCI) and treated with vehicle, 2 nM E2, or 20 nM E2 immediately before measuring reactive oxygen species (ROS) production, bioenergetics, electron transport chain complex (ETC) activities, and β-oxidation of palmitoyl carnitine. Protein expression of oxidative phosphorylation (OXPHOS) complexes was also measured in these mitochondrial samples to determine whether this influenced functional outcomes with respect to sex or injury. While mitochondrial ROS production was affected by CCI in both sexes, there were other sex-specific patterns of mitochondrial injury 24 h following severe CCI. For instance, mitochondria from males were more susceptible to CCI-induced injury with respect to bioenergetics and ETC complex activities, whereas mitochondria from females showed only Complex II impairment and reduced β-oxidation after injury. Neither concentration of E2 influenced ETC complex activities themselves, but 20 nM E2 appeared to uncouple mitochondria isolated from the contralateral cortex in both sexes, as well as the injured ipsilateral cortex of females. These studies highlight the significance of measuring mitochondrial dysfunction in both sexes after TBI and also shed light on another potential neuroprotective mechanism in which E2 may attenuate mitochondrial dysfunction after TBI in vivo.

## 1. Introduction

Traumatic brain injury (TBI) is a debilitating disease that affects millions of people across the United States annually. As more women are coming forward about intimate partner violence, participating in high-impact sports, or enlisting in active military duty, TBI research in women must also increase [1,2,3,4]. An excellent review by Gupte et al. found that 46% of human studies and 55% of animal studies reported females had improved outcomes compared to males after severe TBI [5]. Interestingly, the opposite was found after mild TBI: women tended to have poorer outcomes compared to men, although the animal studies had mixed results [5]. The review contained studies with a broad age range of participants, so it is difficult to determine whether these outcomes could be correlated to the likely abundance of circulating hormones for a particular age group. Traditionally, pre-clinical research has been limited to the use of only males for several reasons, but the most common is the lack of estrus cycling in males, which was thought to reduce some experimental variables [5,6]. Obtaining the menstrual cycle status in women at the time of brain injury is not common practice, so the use of cycling female animals in research should not be a deterrent [5,7]. However complex they may be, there is no question that hormones play a role in either ameliorating or worsening recovery of injury and disease progression. An important example of the effect of sex hormones on brain injury is the use of progesterone to alleviate secondary injury after TBI.

Progesterone was shown to be a promising therapeutic for the treatment of acute TBI as preclinical studies indicated protective effects in the CNS and intestinal mucosa following ischemic and traumatic brain injury [8,9,10,11]. Encouraging results were also seen in Phase II clinical trials; however, Phase III clinical trials failed to see neurocognitive improvements [12,13,14]. Nonetheless, the clinical trials brought up an important point: progesterone is not just a reproductive hormone; it can also be used as a neurotherapeutic. Thus, the role of sex hormones in brain injury should not be overlooked. There is evidence that having 17β-estradiol (E2), the most potent estrogen present during a woman’s reproductive life, present at the time of injury can maintain mean arteriole blood pressure (MAP) in rats acutely after weight drop TBI and that spared MAP is correlated with increased survival [15]. Pretreatment with E2 has also been shown to reduce cell death in ischemic brain injury through increased expression of the anti-apoptotic protein, B-cell lymphoma 2 (bcl-2) [16,17]. Conversely, there is evidence that exposure to E2 during an injury has no effect on cortical tissue sparing, hippocampal degeneration, or microglial activation 7 days post-controlled cortical impact (CCI) brain injury [18]. Alternatively, E2 has also been administered as a treatment after TBI. Studies have shown that an acute supraphysiological dose of E2 post-lateral fluid percussion injury increases ipsilateral hippocampal neuronal survival and decreases neuronal degeneration in both the ipsilateral hippocampus and cortex 24 h post-injury [19]. Another study found subcutaneous delivery of conjugated estrogens 3 days post dynamic cortical deformation injury reduced cell death in the perilesional area, likely due to upregulation of neuronal bcl-2 [20]. Further, Naderi et al. found that E2 treatment reduced brain edema and blood-brain barrier disruption after weight-drop TBI, and this mechanism of neuroprotection is not mediated by E2 receptors [21]. Most available literature explores the role of E2 on total physiological mechanisms, but there is also evidence supporting more specific roles for E2 in alleviating secondary injury following TBI.

Several studies have identified E2 as an antioxidant and have used it to treat secondary brain injury [22,23,24,25]. Culmsee and colleagues observed that the A-ring phenolic hydroxyl group of E2 plays a critical role in the hormone’s ability to scavenge free radicals in a mouse model of ischemic brain injury [26]. Others have reported that E2 may impart neuroprotection by forming a nonphenolic quinol after capturing hydroxyl radicals produced by the Fenton reaction [27]. Shahrokhi et al. found that both physiological and pharmacological concentrations of E2 affected the activity of the endogenous enzymatic antioxidants superoxide dismutase (SOD) and glutathione peroxidase (GPx) after experimental TBI in ovariectomized rats [28]. Specifically, one pharmacological dose of E2 was found to increase SOD activity, while a physiological dose of E2 reduced GPx activity compared to vehicle treatment [28]. Research has shown that antioxidants can be used to reduce oxidative damage and improve functional outcomes after TBI [29,30,31,32,33]. Such findings suggest that the antioxidant properties of E2 have the potential to contribute to the recovery of TBI. A review by Davis and Vemuganti highlighted non-enzymatic antioxidant therapies that improved physiological and functional outcomes following TBI in animal models [34]. Many of the tested therapies had antioxidant mechanisms similar to those possessed by estrogen that were previously discussed, including free radical scavengers and activation of endogenous antioxidants [34]. However, sex differences have also been observed in the treatment of TBI with antioxidant therapies in animal models. For example, the antioxidant therapy Pro-NP^TM^ has been shown to decrease ROS production in male and female mice 4 h post-TBI, but only the male mice were shown to have significantly affected markers of oxidative stress [35]. Thus, sex differences have been seen with antioxidant therapies for TBI, and E2 has antioxidant mechanisms that may help contribute to the development of new therapeutics in the future. Specifically, mitochondria are key regulators of secondary injury following TBI and are major sources of oxidative stress within cells that can be targeted therapeutically to alleviate dysfunction after injury.

Mitochondrial dysfunction is a hallmark of TBI that leads to reduced energy (adenosine triphosphate; ATP) production or ATP consumption, excessive reactive oxygen species (ROS) generation, and poor calcium buffering abilities [36,37,38,39,40,41]. Mitochondrial dysfunction, if allowed to persist, can trigger cell death pathways to worsen long-term functional outcomes after TBI [41,42,43]. This dysfunction has been well-established in male rodent models of TBI, but researchers are still learning about mitochondrial impairment in females. It has been shown by Greco et al. that the total mitochondrial population isolated from the injured cortex of female rats had little/no mitochondrial respiratory dysfunction 24 h after injury, while mitochondria from males did [44]. Our group has recently shown in mice that this is likely due to spared non-synaptic (neuronal and glial origin) mitochondrial function masking impaired synaptic (primarily neuronal) mitochondrial function in females [45]. The cause of these sex differences is still unknown, but one avenue to be explored is the impact of hormones on mitochondrial function, particularly the “neuroprotective” estrogen.

Estrogen actions have been shown to converge on mitochondria to influence their function [46,47]. Traditionally, the most potent estrogen during a woman’s reproductive life, E2, is known to influence mitochondrial function through the regulation of genes involved in glycolysis and other metabolic pathways, oxidative phosphorylation, free radical maintenance, and cellular apoptosis (reviewed by [46]) [16,47,48,49,50,51,52,53]. The impact of E2 on mitochondrial bioenergetics is of particular interest to our group since there is evidence that the removal of endogenous hormone production by ovariectomy reduces mitochondrial respiratory function in the brain (reviewed by [36]). Very few studies have examined the role of E2 directly on mitochondrial functions. However, of the studies available, Borrás et al. found that nanomolar concentrations (0.2 nM–2000 nM) of E2 reduced peroxide production in isolated liver mitochondria [54]. Torres et al. found ex vivo treatment of skeletal muscle mitochondria from OVX rats with 3 nM E2 partially restored ETC Complex I activity compared to vehicle control [22]. A study by Moreno et al. showed 25 µM E2 promoted “uncoupling” of the ATP synthase in isolated liver mitochondria, a mechanism that has been shown to attenuate mitochondrial dysfunction after brain injury with other uncoupling compounds [55,56,57,58]. These aforementioned studies all have different proposed mechanisms for the direct action of estrogen on mitochondrial function, any of which could provide therapeutic potential after TBI, though this has yet to be tested. These present studies hypothesized that the direct administration of E2 with isolated mitochondria would improve functional outcomes after experimental TBI. This hypothesis was tested by measuring mitochondrial ROS production, bioenergetics, electron transport chain (ETC) complex activities, and β-oxidation of palmitoyl carnitine 24 h-post severe CCI in both male and female mice. 

## 2. Methods

### 2.1. Animals and Experimental Design

These studies were approved by the University of Kentucky Institutional Animal Care and Usage Committee (IACUC; PHS Assurance #D16-00217; IACUC Approval #2019-3391) and the United States Veterans Affairs Animal Component of Research Protocol. Additionally, the Division of Laboratory Animal Resources at the University is accredited by the Association for the Assessment and Accreditation for Laboratory Animal Care, International (AAALAC, International), and all experiments were performed within its guidelines. All animal experiments were compliant with ARRIVE guidelines, and experiments were carried out in accordance with the National Institutes of Health Guide for the Care and Use of Laboratory Animals (NIH Publications No. 8023, revised 1978). All experiments were conducted using adult male and female (2–3 months) wild-type C57BL/6 (Jackson Laboratories) mice with average masses of approximately 25 g (male) or 20 g (female). The animals were housed in the same room, five per cage, and maintained in a 14-h light/10-h dark cycle. All animals had access to a balanced diet, and reverse osmosis generated water ad libitum. There was no randomization of treatment groups because, for all experiments, each mitochondrial sample was treated with all concentrations of 17β-estradiol (E2) or vehicle solution. The precise numbers of animals utilized for each experiment, as well as the number of technical replicates, if applicable, are reported in the figure legends.

### 2.2. Estradiol and Vehicle Formulations

The concentrations were selected based on previous literature reporting that 2 nM and 20 nM E2 reduced mitochondrial peroxide production [54]. For the ROS assay, 17β-estradiol (E2; Sigma, Tokyo, Japan, Cat #E8875-1G) was prepared in 100% ethanol. 2 µL of each concentration was added to the well to achieve final concentrations of 2 nM and 20 nM E2 in a total volume of 100 µL. The vehicle for ROS assay was 2% ethanol. For the bioenergetic, ETC complex activities, and β-oxidation assays, E2 was prepared in ethanol first and then diluted with respiration buffer (215 mM mannitol, 75 mM sucrose, 0.1% bovine serum albumin [BSA], 20 mM HEPES, 2 mM MgCl_2_, and 2.5 mM KH_2_PO_4_, pH 7.2) to achieve final concentrations of 2 nM or 20 nM E2 in a total volume of 145 µL. The vehicle for these assays was 3.5% ethanol. For all experiments, vehicle or E2 treatments were added to diluted wells immediately before running the assay so that mitochondria were not exposed to 100% ethanol. Treatments were present for the duration of each assay.

### 2.3. Mouse Model of Traumatic Brain Injury

Controlled cortical impact (CCI) injury procedures were similar to previously published studies [40,58]. In short, mice were first anesthetized with 4.0% isoflurane so their heads could be shaved and then placed into a stereotaxic frame. During the injury procedure, isoflurane was maintained at 2.5% via nose cone. Bupivacaine (Covetrus, Portland, ME, USA, Cat #77614) was injected under the scalp, and then the area was cleaned with an alcohol wipe, followed by an iodine wipe. An incision was made to expose the skull; a ~4 mm diameter craniotomy was performed laterally to the sagittal suture, positioned centrally between bregma and lambda. The skull cap was carefully removed so the dura mater remained intact, and then the stereotaxic frame was transferred to the impactor device (Precision Systems and Instrumentation, Lexington, KY, USA) to receive the CCI. The impactor tip had a diameter of 3 mm, and the impact velocity was set to 3.5 m/s, a depth of 1.00 mm, and a dwell time of 500 ms. A depth of 1.00 mm in a mouse is considered to be a severe injury [37,58]. Following the impact, a ~4 mm^2^ piece of hemostatic dressing was placed over the exposed cortex, and then the incision was closed with staples. The mice were allowed to recover in a clean cage over a heating pad at 37 °C until consciousness was regained. No pain medication was applied after surgery as systemic analgesics of opioid or NSAID classes have been demonstrated to influence the outcome of CCI, which can alter the interpretation of histological and biochemical data. All potentially useful analgesics alter brain receptor function, and many have been reported to be neuroprotective. Any animal exhibiting signs of discomfort (i.e., vocalization, biting, abnormal posture) immediately following surgery is referred to a veterinarian for further evaluation. However, none of the mice in this study showed signs of pain or distress 4 h after surgery. Mice were euthanized 24 h later for mitochondrial isolation. 

### 2.4. Isolation of Mitochondria from Brain Tissue

Mice were rendered unconscious with carbon dioxide (~70% cage volume per minute) and immediately decapitated. The brain was removed, and the cortices were dissected. A 4 mm diameter punch was taken off the cortex ipsilateral to the injury, centered over the site of CCI, containing the injury core and penumbra. An equivalent punch of the contralateral cortex was also taken to serve as the internal, unimpacted control. The tissue was homogenized in a 2 mL isolation buffer (215 mM mannitol, 75 mM sucrose, 0.1% BSA, 20 mM HEPES, 1 mM EGTA, adjusted pH 7.2 with KOH). Total mitochondria were then isolated by Ficoll density gradient ultracentrifugation [59,60,61]. Total protein concentrations of the mitochondrial enriched samples were estimated using the Pierce BCA protein assay kit (Thermo Fisher Scientific, Waltham, MA, USA, Cat #23227) in the Biotek Synergy HTX multi-mode plate reader (Agilent Technologies, Santa Clara, CA, USA). 

### 2.5. Reactive Oxygen Species Production

ROS production was measured in 5 µg of freshly isolated mitochondria under both low and high membrane potential (ΔΨm) conditions, as described previously [59,62,63]. Low ΔΨm is predicted to be less than 100 mV, and high is greater than 160 mV based on the activity of the ATP synthase [37]. Each mitochondrial sample (5 µg) was loaded onto a black 96 well plate so that measurements of oligomycin and FCCP were obtained in duplicate for each concentration of E2 (i.e., 2 nM or 20 nM; *n* = 6/group with 2 technical replicates) or vehicle (2% ethanol). Low ΔΨm was induced by the addition of the mitochondrial uncoupler, FCCP. The FCCP mixture was formulated in respiration buffer to obtain the final concentrations of the following reagents: 2 µM FCCP, 5 mM pyruvate, 2.5 mM malate, 10 µM 2′,7′-dichlorodihydroflurorescein diacetate (DCF-DA; Molecular Probes #D-399, Eugene, OR, USA, 2021), and 30 µL of 20 mg/mL horseradish peroxidase (HRP; Sigma P-6782, 2021, 250 units/mg solid) per 6 mL solution. High ΔΨm was induced by the addition of the ATP synthase inhibitor, oligomycin. The oligomycin mixture was prepared in the buffer to achieve a final concentration of 2.5 µM oligomycin, excluding FCCP. After the addition of 100 µL of either FCCP or oligomycin mixture, the fluorescence was read immediately at 485 ex./528 em. nm and then at 1-min intervals for 40 min in the Synergy HTX microplate reader (BioTek, Winooski, VT, USA) at 37 °C. The relative ROS production at 30 min, the time at which the slope of the line of fluorescent readings across time was linear for all samples, was selected for analysis. Data were normalized to blank wells containing FCCP/oligomycin mixture for each vehicle or E2 concentration and no mitochondria. Mitochondria were then stored at −20 °C for future assessment of ETC complex activities.

### 2.6. Mitochondrial Bioenergetic Measurements

Immediately following isolation, mitochondrial bioenergetics were analyzed on the Seahorse XFe96 Analyzer (Agilent Technologies, Santa Clara, CA, USA), as described previously [40,45,59]. In short, mitochondrial samples were diluted in respiration buffer (125 mM KCl, 0.1% BSA, 20 mM HEPES, 2 mM MgCl_2_, and 2.5 mM KH_2_PO_4_, adjusted to pH 7.2 with KOH) so that 1 µg mitochondrial protein was loaded per well (*n* of 6 with 3–4 technical replicates per sample) and then were treated with vehicle or E2 immediately prior to bioenergetic measures. Oxygen Consumption Rates (OCR) are measured after the addition of substrates, inhibitors, and uncouplers of the mitochondrial Electron Transport Chain (ETC) prepared in respiration buffer without BSA [40,59]. State III respiration, or ATP production-linked respiration, was measured after the addition of pyruvate (5 mM) and malate (2.5 mM), which are Complex I-specific substrates, and ADP (4.3 mM) to activate the ATP synthase. State IV respiration, or proton leak respiration, was measured after the addition of oligomycin (2.5 µM), which inhibits ATP synthase. State V(CI) respiration, or Complex I-driven uncoupled respiration, was measured after the addition of FCCP (4 µM), a mitochondrial uncoupler that carries protons back into the matrix, thus ramping up respiration. Finally, State V(CII) respiration, or Complex II-driven uncoupled respiration, was measured after the addition of the Complex I inhibitor, rotenone (0.8 µM), and the Complex II-specific substrate, succinate (10 mM). 

### 2.7. Electron Transport Chain Complex Activities

Assessment of mitochondrial ETC complex activities in reserved mitochondrial samples was performed using the Seahorse XFe96 Analyzer (Agilent Technologies, Santa Clara, CA, USA) as described previously [59,64]. Briefly, 0.3 µg mitochondrial protein in Mir05 buffer (0.5 mM EGTA, 3 mM MgCl_2_, 60 mM lactobionic acid, 20 mM taurine, 10 mM KH_2_PO_4_, 20 mM HEPES, 110 mM sucrose, 0.1% BSA pH 7.1 with KOH) were loaded per well. The OCR measurements were carried out using Seahorse after slowly adding the top-up solution containing alamethicin (35 µg/mL), nicotinamide adenine dinucleotide (NADH; 3.5 mM), cytochrome c (17.52 µM), and either vehicle or E2 (*n* = 4–6/group with 3 technical replicates per sample). Remaining substrates and inhibitors of the ETC were prepared in Mir05 without BSA and loaded into the injection ports as follows: (A) rotenone (0.8 µM) and succinate (10 mM), (B) antimycin A (1 µM), (C) ascorbate (20 mM) and *N*,*N*,*N*′,*N*′-tetramethyl-*p*-phenylenediamine (TMPD; 5 mM), (D) sodium azide (549.3 mM). Complex I activity was calculated by subtracting the Antimycin A reading from the baseline (NADH) reading. Complex II activity was measured by subtracting the Antimycin A reading from the rotenone/succinate reading. Complex IV activity was calculated by subtracting the azide reading from the ascorbate/TMPD reading. 

### 2.8. OXPHOS Protein Expression

The relative expression of proteins involved in the mitochondrial oxidative phosphorylation (OXPHOS) system was carried out using Western blot analysis, as follows. Mitochondrial samples were not exposed to E2 for Western blot analysis since these preparations lack the machinery to influence transcriptional pathways related to OXPHOS protein expression. Reserved mitochondrial samples were prepared in XT sample buffer (Bio-Rad, Hercules, CA, USA, Cat #1610791) and incubated at room temperature (~25 °C) for 20 min. 10 µg of each sample were loaded onto a 4–12% Bis-Tris gel (Bio-Rad, Hercules, CA, USA, Cat #3450125) and subjected to electrophoresis at 150V. Proteins were transferred to a polyvinylidene difluoride (PVDF) membrane using the Bio-Rad Trans-blot Turbo Transfer system, according to the manufacturer’s instructions. Following the transfer, the membranes were incubated in blocking buffer (5% milk in TBST wash buffer [1× tris-buffered saline and 0.1% Tween^®^ 20]) for one hour at room temperature. Membranes were then briefly rinsed with wash buffer and then incubated with primary antibody (1:1000 Total OXPHOS Rodent WB Antibody Cocktail, Abcam, Cambridge, UK, #ab110413 and 1:1000 TOM20, Cell Signaling, Danvers, MA, USA #42406) in blocking buffer (3% BSA) overnight at 4 °C with slow, continuous rocking. Membranes were washed 3 times for 5 min with wash buffer and then incubated with secondary antibody (1:15,000 goat anti-mouse #926-68070 and 1:20,000 goat anti-rabbit #926-32211, LI-COR, Lincoln, NE, USA) for one hour at room temperature. The membranes were washed 3 times for 5 min with wash buffer and then imaged on the Odyssey CLx imager. The mean gray volume of each band was quantified using ImageJ software (National Institutes of Health, Bethesda, MD, USA), and protein expression for each OXPHOS band was normalized to TOM20. 

### 2.9. β-Oxidation Respiratory Measurement

Beta oxidation of palmitoyl DL-carnitine (Cayman Chemical Company, Ann Arbor, MI, USA Item #11095) by mitochondria was measured on the Seahorse XFe96 Analyzer (Agilent Technologies, Santa Clara, Ca, USA). To ensure sufficient mitochondrial concentrations were isolated, two cortical punches from two mice (within the same sex and injury group) were pooled together for each sample. A total of 2 µg of mitochondrial protein in respiration buffer was loaded per well (*n* = 6/group with 3–4 technical replicates per sample) and treated with vehicle or E2 immediately prior to measurement. The substrates were prepared in respiration buffer without BSA and loaded into the injection ports as follows: (A) palmitoyl l-carnitine (40 µM) and malate (2.5 mM), (B) ADP (4.3 mM).

### 2.10. Statistical Analysis

Power analysis was performed using G*Power software (Germany, v.3.1.9.7) with the following assumptions: α = 0.05, 1 − β = 0.80, and a standard deviation of 10% with State III respiration as the primary outcome measure. The resulting sample size was determined to be *n* of 6 mice/group. All analyses were performed using GraphPad Prism (GraphPad Software Inc., La Jolla, CA, USA). All data from males and females were analyzed separately. The Shapiro-Wilk test was employed to assess normality. For each experiment, paired *t*-tests were performed between vehicle-treated mitochondria from the ipsilateral and contralateral cortices to assess the injury effect. Since all mitochondrial samples were treated with all treatments, data within each cortical region were analyzed by one-way repeated measures analysis of variance (ANOVA), assuming sphericity, to assess the treatment effect. Sidak’s multiple comparisons test was applied when appropriate. For data containing missing values, a mixed-effects model was employed for analysis and is stated in the figure legend.

## 3. Results

### 3.1. ROS Production

**Figure 1:**
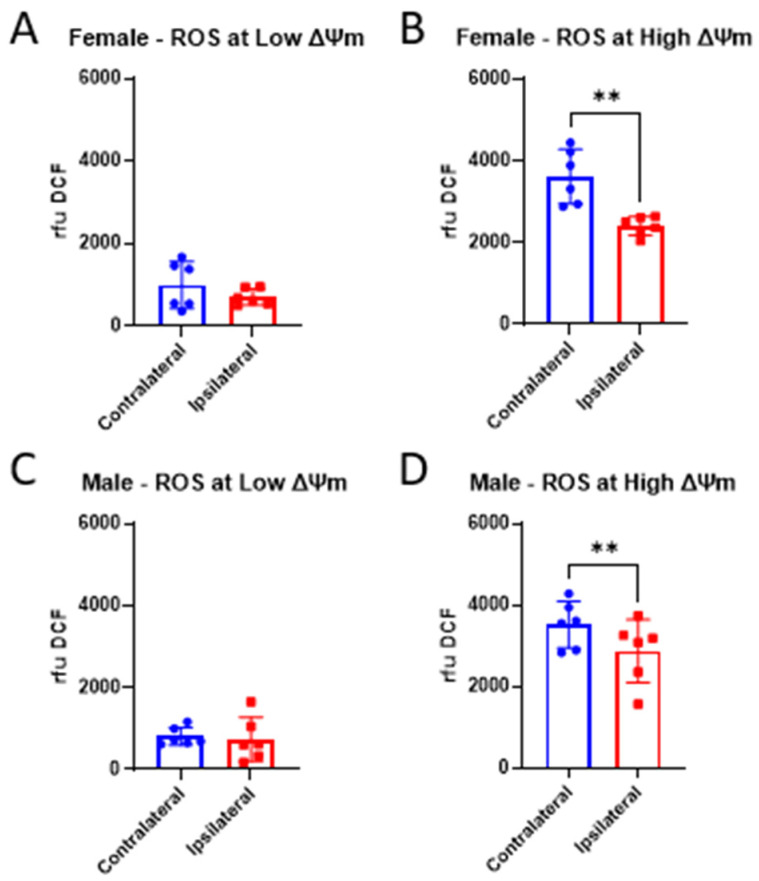
Assessment of injury on reactive oxygen species (ROS) production in cortical mitochondria from male and female mice 24 h post-CCI. Total mitochondria were isolated from the cortices ipsilateral to the CCI, as well as contralateral to the CCI, to serve as the uninjured control in female (**A**,**B**) and male (**C**,**D**) mice. Low membrane potential (ΔΨm), a state in which ROS production is low, was induced by FCCP, and high ΔΨm, a state in which ROS production is high, was induced by oligomycin. ROS production is represented as the mean relative fluorescence of DCF ± SD; *n* of 6/group. These data were collected from vehicle-treated mitochondria and were analyzed by one-tailed paired *t*-test to assess the injury effect between the ipsilateral and contralateral cortices. ** *p* < 0.01.

**Figure 2:**
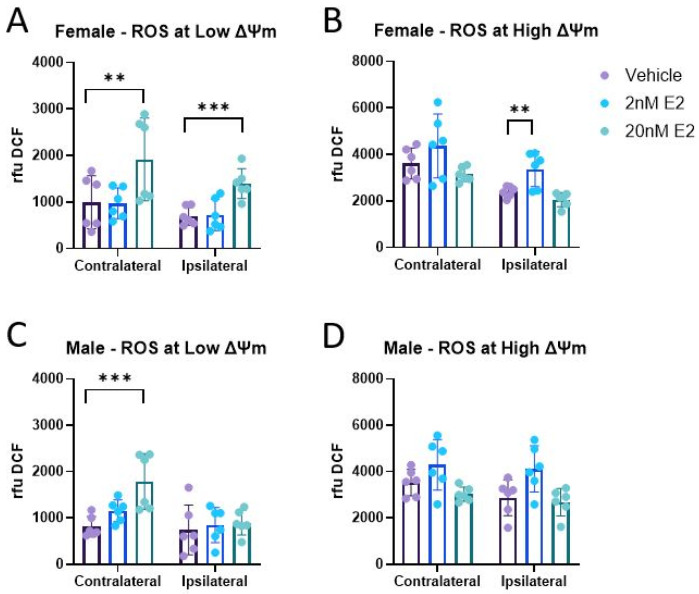
Reactive oxygen species (ROS) production in cortical mitochondria from male and female mice in the presence of 17β-estradiol 24 h post-CCI. Total mitochondria were isolated from the cortices ipsilateral to the CCI, as well as contralateral to the CCI, to serve as the uninjured control in female (**A**,**B**) and male (**C**,**D**) mice. Low membrane potential (ΔΨm), a state in which ROS production is low, was induced by FCCP, and high ΔΨm, a state in which ROS production is high, was induced by oligomycin. ROS production is represented as the mean relative fluorescence of DCF ± SD; *n* of 6/group. Each cortical region was analyzed by repeated measures of one-way ANOVA to assess treatment effects with Sidak’s multiple comparisons versus Vehicle, where appropriate. ** *p* < 0.01, *** *p* < 0.001.

Increased oxidative stress has been shown to be a consequence of traumatic brain injury. Mitochondria themselves are major sources of ROS production within the cell, which is exacerbated after injury. Previous work has shown that direct administration of 17β-estradiol (E2) to isolated mitochondria reduced peroxide production [54]. Here, we measured ROS production 24 h post-CCI in the presence of E2 at both low and high mitochondrial membrane potential (ΔΨm), states in which mitochondria produce low or high levels of ROS, respectively. At low ΔΨm, there were no significant differences in ROS production between mitochondria from the injured ipsilateral cortex compared to that of the unimpacted contralateral cortex in either sex (Figure 1A,C). At high ΔΨm, though, ROS production from mitochondria ipsilateral to the injury was significantly reduced by CCI compared to mitochondria from the contralateral cortex in both sexes (Figure 1B,D). This decrease in ROS production is likely a result of CCI-induced disruption of mitochondrial membranes, making oligomycin less effective in generating high ∆Ψ. Additional analysis within each cortical region showed 20 nM E2 increased ROS production in both the ipsilateral and contralateral cortices compared to the vehicle (Figure 2A). At high ΔΨm in females, 2 nM E2 increased ROS production in mitochondria from the ipsilateral cortex compared to the vehicle (Figure 2B). Similar to females, there was also a significant treatment effect in males at low ΔΨm in which 20 nM E2 increased ROS production compared to vehicle in the contralateral cortex (Figure 2C); however, there were no treatment effects detected at high ΔΨm (Figure 2D). 

### 3.2. Bioenergetics

**Figure 3:**
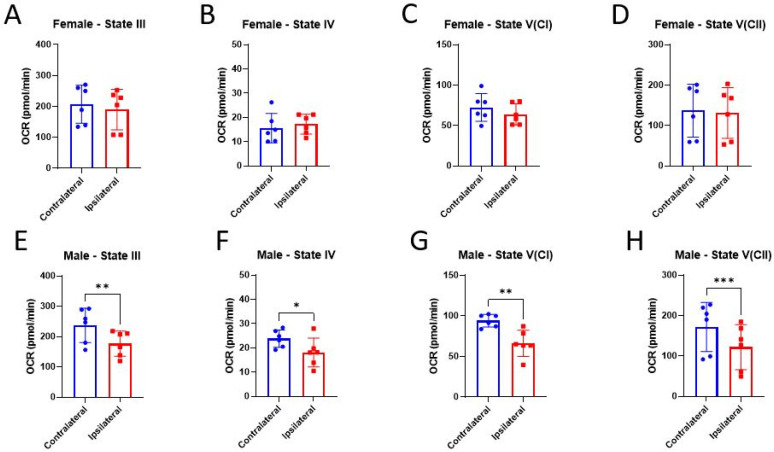
Assessment of injury on mitochondrial bioenergetics 24 h post-CCI. Total mitochondria were isolated from the cortices ipsilateral to the CCI, as well as contralateral to the CCI, to serve as the uninjured control in female (**A**–**D**) and male (**E**–**H**) mice. Data are represented as mean oxygen consumption rate (OCR) ± SD; *n* of 6/group. State III is measured after the addition of pyruvate, malate, and ADP. State IV was measured after the addition of oligomycin. State V(CI) was measured after the addition of FCCP. State V(CII) was measured after the addition of rotenone and succinate. These data were collected from vehicle-treated mitochondria and were analyzed by one-tailed paired *t*-test to assess the injury effect between the ipsilateral and contralateral cortices. * *p* < 0.05, ** *p* < 0.01, *** *p* < 0.001.

**Figure 4:**
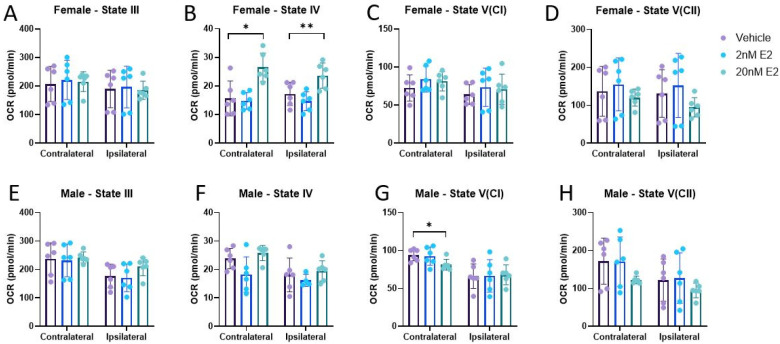
Mitochondrial bioenergetics in the presence of 17β-estradiol 24 h post-CCI. Total mitochondria were isolated from the cortices ipsilateral to the CCI, as well as contralateral to the CCI, to serve as the uninjured control in female (**A**–**D**) and male (**E**–**H**) mice. Data are represented as mean oxygen consumption rate (OCR) ± SD; *n* of 6/group. State III is measured after the addition of pyruvate, malate, and ADP. State IV was measured after the addition of oligomycin. State V(CI) was measured after the addition of FCCP. State V(CII) was measured after the addition of rotenone and succinate. Each cortical region was analyzed by repeated measures of one-way ANOVA to assess treatment effects with Sidak’s multiple comparisons versus Vehicle, where appropriate, with one exception. State IV respiration from the ipsilateral cortex was analyzed using a mixed-effects model due to missing values. * *p* < 0.05, ** *p* < 0.01.

Our group, as well as others, have shown that total mitochondria from females are resilient to CCI-induced bioenergetic dysfunction, while mitochondria from males are not [44,45]. There is also evidence that E2 directly influences bioenergetic functions in mitochondria from uninjured skeletal muscle [22]. Here, we measured total mitochondrial bioenergetics in male and female mice 24 h post-CCI in the presence of ex vivo E2. Consistent with the current literature, mitochondria from the ipsilateral cortex of females did not show any bioenergetic impairments compared to the un-impacted, contralateral control (Figure 3A–D). In males, mitochondria isolated from the cortex ipsilateral to the CCI had significantly impaired respiration across all respiration states compared to the contralateral control (Figure 3E–H). Interestingly, 20 nM E2 increased State IV respiration in mitochondria from both cortical regions of female mice (Figure 4B), but no other treatment effects were detected. The only treatment effect detected in males was at State V(CI), in which 20 nM E2 reduced respiration in mitochondria isolated from the contralateral cortex (Figure 4G).

### 3.3. ETC Complex Activities 

**Figure 5:**
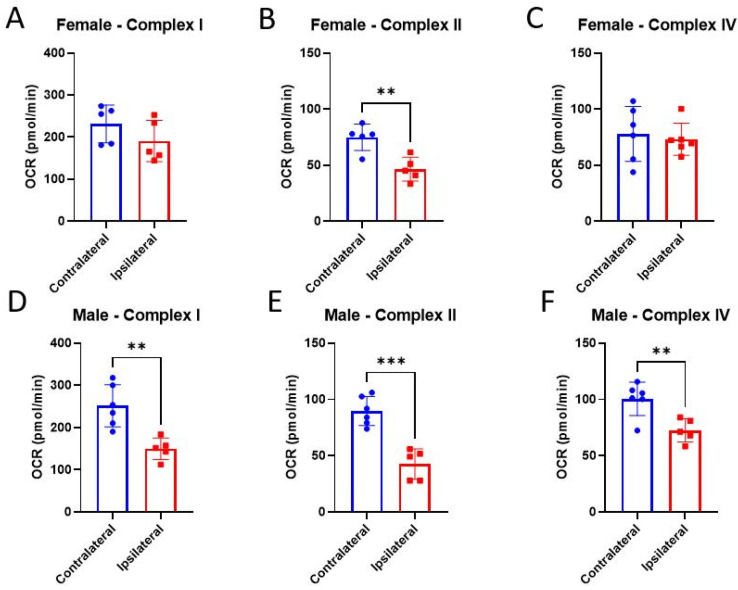
Assessment of injury on mitochondrial electron transport chain (ETC) complex activities 24 h post-CCI. Total mitochondria were isolated from the cortices, both ipsilateral and contralateral, to the CCI in female (**A**–**C**) and male (**D**–**F**) mice. Data are represented as mean oxygen consumption rate (OCR) ± SD; *n* of 4–6/group. Complex I activity was calculated by subtracting the OCR of the antimycin A reading from the baseline OCR reading. Complex II activity was calculated by subtracting the antimycin A reading from the rotenone/succinate reading. Complex IV was calculated by subtracting the sodium azide OCR reading from the ascorbate/TMPD OCR reading. Vehicle data were analyzed using a one-tailed unpaired *t*-test to assess the effect of injury on the ipsilateral and contralateral cortices. ** *p* < 0.01, *** *p* < 0.001.

**Figure 6:**
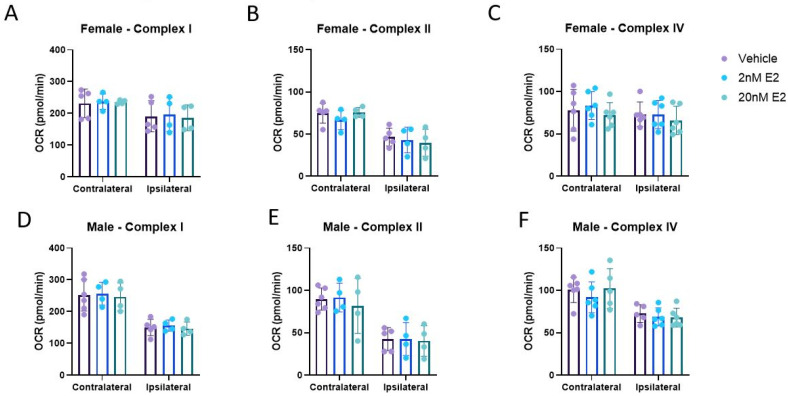
Mitochondrial electron transport chain (ETC) complex activities in the presence of 17β-estradiol 24 h post-CCI. Total mitochondria were isolated from the cortices ipsilateral to the CCI, as well as contralateral to the CCI, to serve as the uninjured control in female (**A**–**C**) and male (**D**–**F**) mice. Data are represented as mean oxygen consumption rate (OCR) ± SD; *n* of 4–6/group. Complex I activity was calculated by subtracting the OCR of the antimycin A reading from the baseline OCR reading. Complex II activity was calculated by subtracting the antimycin A reading from the rotenone/succinate reading. Complex IV was calculated by subtracting the sodium azide OCR reading from the ascorbate/TMPD OCR reading. Vehicle data were analyzed using a one-tailed unpaired *t*-test to assess the effect of injury on the ipsilateral and contralateral cortices. Each cortical region was analyzed by repeated measures of one-way ANOVA using a mixed-effects model to assess treatment effects, with one exception. Complex IV activity for both the ipsilateral and contralateral cortical regions of females (**C**) were analyzed by repeated measures of one-way ANOVA to assess treatment effects.

Mitochondrial bioenergetics are invaluable measurements that allow us to detect functional mitochondrial impairment after CCI. However, it is not always clear whether this dysfunction is upstream of the ETC or within it. It is particularly important to measure ETC activities in both sexes since mitochondria from females appear to have little to no bioenergetic deficits after CCI. Here, we measured ETC complex activities directly in a single well system to understand how CCI-induced damage to these complexes contributes to the observed mitochondrial bioenergetic dysfunction. Additionally, we sought to determine whether E2 directly interacts with the ETC complexes to influence function. There was only a significant injury effect in Complex II activity after CCI in mitochondria from females (Figure 5B). Consistent with the bioenergetic literature, the ipsilateral, injured mitochondria from males showed impairments in all three complexes measured compared to the contralateral cortex (Figure 5D–F). E2 had no observed effects on complex activities in either cortical region in either sex (Figure 6A–F). 

### 3.4. OXPHOS Protein Expression

**Figure 7:**
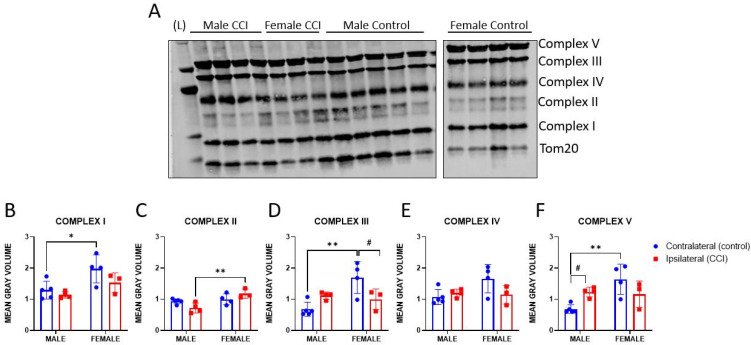
Assessment of injury on protein expression of complexes involved in the mitochondrial oxidative phosphorylation (OXPHOS) process 24 h post-CCI. Mitochondria isolated from both the ipsilateral (injured) and contralateral (uninjured control) cortices of male and female mice were subjected to Western blot analysis (**A**) to determine the relative expression of Complex I (**B**), Complex II (**C**), Complex III (**D**), Complex IV (**E**), and Complex V (**F**). The mean gray volume of each band was normalized to Tom20. Values are represented as mean ± SD: Male Contralateral *n* of 5, Male Ipsilateral *n* of 4, Female Contralateral *n* of 4, Female Ipsilateral *n* of 3. Data were analyzed by two-way ANOVA with Sidak’s multiple comparisons, where appropriate. Compared to males of the respective injury group: * *p* < 0.05; ** *p* < 0.01. Compared to contralateral control: ^#^ *p* < 0.05.

CCI injury has been shown to cause extensive mitophagy and cell death in male rodent models of injury, though not much is known about these processes in females [65,66,67,68]. To determine whether the functional changes in mitochondrial bioenergetics and ETC complex activities correlate to these processes, we measured protein expression of the five proteins involved in the oxidative phosphorylation (OXPHOS) system (i.e., Complex I-IV and ATP synthase [Complex V]) 24 h after CCI in male and female mice. There was little effect of injury on OXPHOS protein expression, with two exceptions. Complex III expression decreased 24 h after injury in females (Figure 7D), and Complex V expression increased in males after injury (Figure 7F). Interestingly, Complex I, III, and V expressions were significantly higher in mitochondria isolated from the contralateral, unimpacted cortex of females relative to the contralateral cortex of males (Figure 7B,D,F). The expression of these proteins also appeared to decrease after injury in females, but this was not statistically significant in any case. Complex II expression, however, was significantly higher in mitochondria isolated from the ipsilateral, injured cortex of females relative to that of males (Figure 7C).

### 3.5. Beta-Oxidation of Palmitoyl Carnitine

**Figure 8:**
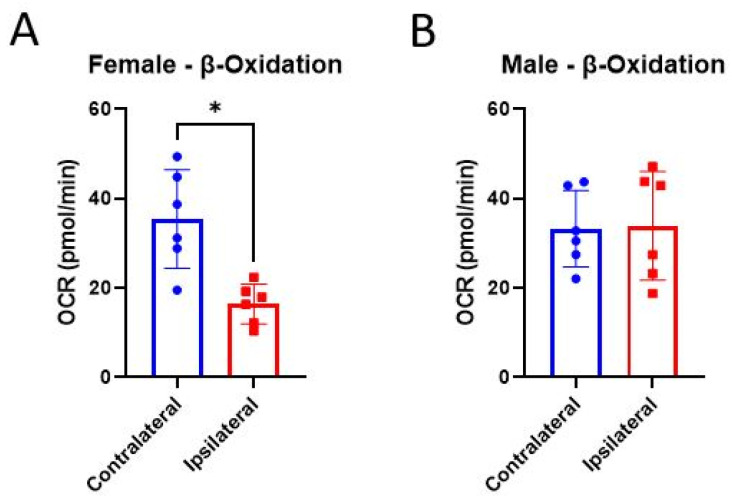
Assessment of injury of mitochondrial β-oxidation of palmitoyl carnitine 24 h post-CCI. Total mitochondria were isolated from the cortices, both ipsilateral and contralateral, to the CCI in female (**A**) and male (**B**) mice. Data are represented as mean oxygen consumption rate (OCR) ± SD; *n* of 6/group. β-oxidation was measured after the addition of palmitoyl carnitine, malate, and ADP. Vehicle data were analyzed using a one-tailed paired *t*-test to assess the effect of injury on the ipsilateral and contralateral cortices. * *p* < 0.05.

**Figure 9:**
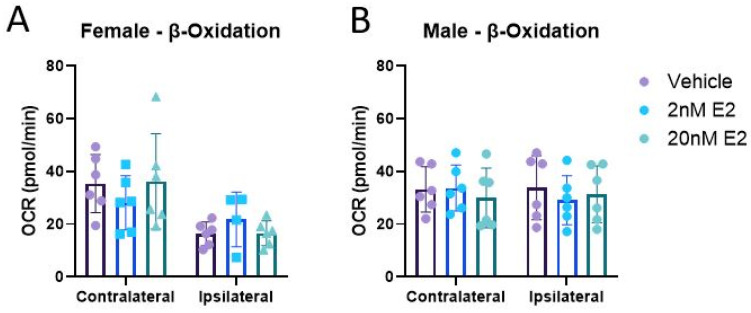
β-oxidation of palmitoyl carnitine by isolated mitochondria in the presence of 17β-estradiol 24 h post-CCI. Total mitochondria were isolated from the cortices, both ipsilateral and contralateral, to the CCI in female (**A**) and male (**B**) mice. Data are represented as mean oxygen consumption rate (OCR) ± SD; *n* of 6/group. β-oxidation was measured after the addition of palmitoyl carnitine, malate, and ADP. Each cortical region was analyzed by repeated measures of one-way ANOVA to assess treatment effects.

Since glucose is the brain’s preferred metabolic substrate, much of the present literature regarding metabolic perturbations after TBI has been focused on oxidative glucose metabolism [69,70,71]. Alternative substrates can be administered to bypass hypometabolism after TBI and promote recovery [44,72,73]. These strategies can be of therapeutic benefit, but they also give a hint as to what mechanisms upstream of the ETC may be impaired within mitochondria after brain injury. Additionally, it has been shown that E2 plays a role in upregulating the uptake, mitochondrial transport, and oxidation of fatty acids in skeletal muscle (reviewed by [74]). Here, we measured mitochondrial respiration in the presence of palmitoyl carnitine to assess CCI-induced impairments in β-oxidation and whether E2 affects this process. Mitochondria from the ipsilateral cortex of females had significantly impaired OCR compared to their unimpacted, contralateral controls (Figure 8A), while, interestingly, that of males showed no injury effect (Figure 8B). No treatment effects were detected in either cortical region or sex (Figure 9). 

## 4. Discussion/Conclusions

Estrogen actions have been shown to converge on mitochondria to influence their function primarily through genomic mechanisms, such as by promoting glycolysis and TCA function, increasing expression of proteins critical for oxygen phosphorylation, and increasing defense against free radicals [46]. This is especially important after TBI because mitochondrial dysfunction is a key driver of secondary injury. Few studies have explored the direct influence of estrogen on mitochondrial functions, and no studies have explored this in the context of brain injury. Of the studies available, estrogen was reported to reduce mitochondrial peroxide production, improve bioenergetics, and uncouple the ATP synthase [22,54,55]. Each of these direct actions could contribute to the apparent “mitoprotection” in females after injury, so these studies were performed to explore the direct actions of the most potent estrogen, 17β-estradiol (E2), on mitochondrial functions after experimental TBI in mice. The two main findings from this present study were: 1. Mitochondria from females have a different pattern of injury after CCI compared to males, and 2. E2 appeared to uncouple mitochondria. 

The first main finding is important because it corroborates the current literature showing mitochondria from females appear protected from CCI-induced bioenergetic dysfunction 24 h after severe injury (Figure 3) [44,45]. In these present studies, however, we went a step further to examine additional measures of mitochondrial function and found mitochondria from males had impaired ETC Complex I, II, and IV activities, while mitochondria from females only had impaired Complex II activity (Figure 5). This is a novel finding for the TBI field, though a study conducted in naïve male and female mice showed that gonadal hormones regulate Complex I-driven respiration but not Complex II-driven respiration [51]. This could imply that Complex II is more susceptible to injury than Complex I in females. However, we found that protein expression of Complex II was one of the only OXPHOS complexes that was not significantly higher in the contralateral cortex of females relative to males (Figure 7). Further, Complex II expression in females did not appear to decrease after injury like the other complexes. This could imply that increased protein expression of Complex I can counter its loss after injury and result in spared bioenergetic function, while Complex II may be resilient to degradation after injury but still susceptible to functional impairment after CCI. Mitochondria from females also showed impaired β-oxidation of palmitoyl carnitine after injury, while that of males did not (Figure 7). Together, this data suggests that mitochondria from females are not protected from injury like the literature has implied, but that females may have a different pattern of injury than males. For instance, mitochondrial bioenergetics indicated that females were protected from injury while males were not. Further investigation did indeed reveal preserved mitochondrial bioenergetics and the majority of ETC functions in females; this was not seen in males. However, mitochondria from females had significantly impaired β-oxidation after injury, while males did not. These findings could suggest that mitochondrial injury in females is upstream of the ETC, while injury in males is localized to the ETC. One limitation of this study is that mitochondrial function was only assessed 24 h after injury. This time point was selected based on previous literature reporting sex differences in mitochondrial dysfunction after severe CCI in rodents [44,45]. The time course of mitochondrial dysfunction is well-established in male rodent models of TBI; however, the studies by Kalimon et al. and Greco et al. suggest that females have different metabolic profiles after TBI [44,45,75,76]. This information is crucial to know when determining the proper time of mitochondrial-targeted therapeutic intervention after TBI [44,73,75]. Interestingly, mild mitochondrial uncoupling has been shown to be therapeutically relevant to treating brain injury, which ties into the second main finding of this study [56,57,58,76].

Since mitochondria were isolated and then treated with E2 immediately before performing each experiment, we can effectively rule out the possibility that E2 is acting through classical genomic pathways or other cellular signaling cascades in this preparation. Based on the lack of treatment effects detected in ETC complex activities (Figure 6), it does not appear that E2 is acting on any of the mitochondrial complexes directly to influence function. Instead, it appears as though E2 is mildly uncoupling mitochondria based on the observed increases in State IV respiration (Figure 4B) and ROS production (Figure 2A,C) after treatment with 20 nM E2.

State IV respiration is measured after the addition of oligomycin, an inhibitor of ATP synthase, and is often called “proton-leak-driven respiration” [40,58,77]. A study by Moreno et al. found pharmacological concentrations (25 µM) of E2 caused intrinsic uncoupling of F_0_ and F_1_ subunits of ATP synthase in an ATP concentration-dependent manner [55]. This intrinsic uncoupling of ATP synthase has been more recently described as a c-subunit leak channel, though this is reportedly induced by calcium overload or low ATP levels [78,79,80]. Whether E2 induces the formation of the c-subunit leak channel has not been explored; however, there were no E2-induced changes in State III (ATP production linked) respiration to suggest this as the mechanism of uncoupling. Additionally, E2 has been shown to intercalate into the mitochondrial membrane to affect electron transfer at Complex I and sodium-dependent calcium efflux [22,81,82]. This could suggest that E2 interferes with electron handling within the ETC to promote inefficient respiration and increase ROS production in membrane potential (ΔΨm) conditions where ROS is typically low (Figure 2). Alternatively, E2′s role in inhibiting calcium efflux from mitochondria could influence permeability transition pore opening, although additional studies are required [81,82]. These aforementioned studies, in conjunction with the data presented in this manuscript, suggest that the structure of E2 itself drives the functional changes seen in isolated mitochondria.

The majority of E2 treatment effects were observed in mitochondria from the contralateral, unimpacted cortex of both sexes, as well as the ipsilateral cortex of females. Mitochondria isolated from the contralateral cortex are effectively uninjured, meaning the ETC is well coupled to ATP synthesis, a process that can be uncoupled, which is likely why there was increased ROS production (Figure 2A,C) and State IV respiration (Figure 4B) in the contralateral cortex after 20 nM E2 administration. These uncoupling effects by E2 would likely not be detected in mitochondria from the injured ipsilateral cortex because they are already leaky from the CCI. This is shown best in the context of ROS production at high membrane potential induced by oligomycin. After the injury, ROS production decreased under high membrane potential conditions, which could have been a result of increased leak from CCI, causing oligomycin to be ineffective in generating a high enough membrane potential to generate ROS (Figure 1C and Figure 2C).

The brain uses 20% of the body’s metabolic energy, so it is no surprise that cerebral metabolism is disrupted after TBI [44,83,84,85]. However, not many studies have explored sex differences in adult cerebral metabolism after TBI. Wettervik and colleagues, though, have shown men had worsened brain energy metabolism associated with mitochondrial dysfunction compared to women after severe TBI [86]. The study by Greco et al. highlighted the importance of measuring brain mitochondrial function in both sexes after administration of different metabolic substrates [44]. The ketone body, β-hydroxybutyrate, for instance, improved mitochondrial function after TBI in males but actually exacerbated mitochondrial dysfunction in females [44]. There is evidence that E2 promotes β-oxidation of fatty acids in skeletal muscle through increased gene expression of proteins involved in this process [74]. It is unknown whether E2 would have any direct effects on the mitochondrial processing of fatty acids like palmitoyl carnitine, but based on our data, it appears that it does not. Even so, a distinct sex difference was observed after experimental TBI. Mitochondria from female mice showed impairments in β-oxidation of palmitoyl carnitine after CCI (Figure 7A), while mitochondria from males did not (Figure 7B). Notably, in addition to glycolysis, astrocytes also metabolize fatty acids to regulate lipid homeostasis and protect neurons from lipid damage during times of increased activity [87,88,89]. Villapol and colleagues have shown that male rats had a heightened astrocytic response acutely following CCI compared to females [90]. Perhaps an enhanced astrocytic response and, thus, higher rates of β-oxidation are contributing to the lack of injury effect in mitochondrial respiration driven by palmitoyl carnitine from males seen in these present studies (Figure 7B). Alternatively, data from the skeletal muscle field have shown that females consume fewer supporting substrates (e.g., carbohydrate or protein metabolites) during fatty acid oxidation compared to males [91,92,93]. This suggests that females require less or different supporting substrates to have effective fatty acid oxidation, which may support the deficits observed in these present studies. However, it is unclear whether there are sex differences in supporting substrate utilization during mitochondrial fatty acid oxidation in the brain.

To summarize, after CCI, we found mitochondria from males had impaired bioenergetic function and reduced activities in all ETC complexes measured, while females had only reduced Complex II activity and impaired β-oxidation of palmitoyl carnitine. This data suggests that TBI-induced mitochondrial injury is more localized to the oxidative phosphorylation process in males, while impairments in females may be upstream of the ETC or more prominent in certain cell types. It appears as though the structure of E2 itself is providing the direct effects observed in these present studies, although exogenous E2 administration does not appear to provide direct therapeutic benefit to mitochondria after CCI. It is likely that the majority of the “mitoprotective” effects of E2 are through the indirect genomic mechanisms or cellular signaling cascades in which E2 promotes higher mitochondrial respiratory capacity and reduces oxidative stress after injury. This study is important to the TBI field as a whole as it identifies different mechanisms of mitochondrial injury between the sexes that can each be targeted to improve recovery after brain injury in a sex-specific manner.

## Data Availability

Data will be made available by the authors upon reasonable request.

## Abbreviations

ΔΨm	mitochondrial membrane potential
ATP	adenosine triphosphate
Bcl-2	B-cell lymphoma 2
CCI	controlled cortical impact
DCF-DA	2′,7′-dichlorodihydroflurorescein diacetate
E2	17β-estradiol
ETC	electron transport chain
GPx	glutathione peroxidase
MAP	mean arteriole pressure
NADH	nicotinamide adenine dinucleotide
OCR	oxygen consumption rate
OXPHOS	oxidative phosphorylation
ROS	reactive oxygen species
SOD	superoxide dismutase
TBI	traumatic brain injury
TMPD	*N*,*N*,*N*′,*N*′-tetramethyl-*p*-phenylenediamine
